# Quantitative microglia analyses reveal diverse morphologic responses in the rat cortex after diffuse brain injury

**DOI:** 10.1038/s41598-017-13581-z

**Published:** 2017-10-16

**Authors:** Helena Morrison, Kimberly Young, Mahir Qureshi, Rachel K. Rowe, Jonathan Lifshitz

**Affiliations:** 1 University of Arizona, College of Nursing, 1305 N. Martin Ave, P.O. Box 210203, Tucson, AZ 85721 USA; 2 Arizona State University, Barrett’s Honors College, 698 E Apache Blvd, Tempe, AZ 85281 USA; 3 University of Arizona, College of Medicine – Phoenix, 425 N 5th Street, Phoenix, AZ 85004 USA; 4 Barrow Neurological Institute at Phoenix Children’s Hospital, 1919 E. Thomas Rd., Phoenix, AZ 85016 USA

## Abstract

Determining regions of altered brain physiology after diffuse brain injury is challenging. Microglia, brain immune cells with ramified and dynamically moving processes, constantly surveil the parenchyma for dysfunction which, when present, results in a changed morphology. Our purpose was to define the spatiotemporal changes in microglia morphology over 28 days following rat midline fluid percussion injury (mFPI) as a first step in exploiting microglia morphology to reflect altered brain physiology. Microglia morphology was quantified from histological sections using Image J skeleton and fractal analysis procedures at three time points and in three regions post-mFPI: impact site, primary somatosensory cortex barrel field (S1BF), and a remote region. Microglia ramification (process length/cell and endpoints/cell) decreased in the impact and S1BF but not the remote region (p < 0.05). Microglia complexity was decreased in the S1BF (p = 0.003) and increased in the remote region (p < 0.02). Rod-shaped microglia were present in the S1BF and had a 1.8:1.0 length:width ratio. An in-depth quantitative morphologic analysis revealed diverse and widespread changes to microglia morphology in the cortex post-mFPI. Due to their close link to neuronal function, changes in microglia morphology, summarized in this study, likely reflect altered physiology with diverse and widespread impact on neuronal and circuit function.

## Introduction

Traumatic brain injury (TBI) impacts the lives of over 2.2 million children and adults each year in the United States alone^1,2^. The milder end of the TBI severity spectrum has an unknown incidence, often falling into the lexicon of concussion, and accounts for 70–90% of all TBIs. Although most people survive mild TBI, patient morbidities are diverse and related symptoms depend on the individual, impact site, impact magnitude, and the level of acute care provided at the time of injury. The typical closed-skull impacts of mild TBI are diffuse injuries associated with acceleration and deceleration of the brain, leading to the widespread pathology and broad morbidities that can be debilitating, life-long, and life changing^2^.

Pre-clinical research in rodent models has clarified mechanisms of brain injury after concussion. The mechanical forces of the primary injury disrupt cell membranes^3^, stretch axons, deform tissue, disrupt ion movement^4^, release neurotransmitters, and deplete brain energy stores as cells compensate and respond to the insult(s)^5,6^. From there, secondary injury processes can exacerbate the primary injury to spur waves of neurovascular pathology, including glial dysfunction, cell edema, and inflammatory responses from microglia and astrocytes^6^. In contrast to focal TBI, diffuse brain injury is devoid of gross histological changes, such as cavitation, contusion, and cell death, but rather shows cell atrophy and the accumulation of proteins in axonal swellings^7,8^. Consequently, the full extent of this diffuse and microscopic injury remains challenging to detect in both pre-clinical and clinical research^9^. For this manuscript, the objective quantification of microglia morphology adds essential tools to detect and distinguish diverse morphologies, which may reflect persistent neuropathology across the cortex after diffuse TBI.

Microglia, brain immune cells active in the inflammatory response, continuously surveil brain neurochemistry. In fact, microglia assess the brain every few hours through repeated morphological transformations that include dynamic and active process movements^10^. Neurons can direct microglia surveillance functions during homeostasis and in response to injury through soluble factors that mediate microglia receptor-neuronal ligand interactions^11–15^. In turn, microglia influence neuronal function and maintain neural networks by releasing cytokines (i.e. TNFα^16,17^ and IL-1β) and prostaglandins, as well as through complement mediated phagocytosis (relevant reviews, refs^18–21^). Bi-directional microglia and neuron communication is constant and coordinated, but yet to be fully articulated, especially in the context of injury. However, it is clear that changes in neuronal function or activity, including neuronal dysfunction, initiate immediate transformations in microglia morphology^13–15^. After severe TBI or stroke that leads to cell death and cavitation, microglial responses include morphologic changes, observed as pronounced de-ramification and amoeboid shaped cells, reminiscent of systemic/circulating macrophages^22^. In contrast, diffuse brain injury spares tissue and leaves neurons initially as dysfunctional, with evidence for cellular edema and/or atrophy. In parallel, microglial morphologic response can be mild to robust and regionally dependent, even across the cortex, whereas other regions appear unaffected. Because microglial activity serves as an endogenous sensor for brain disturbance, sensitive and quantitative methods to detect subtle microglia responses to injury can further define regions of unrecognized pathology.

We previously reported on a stark morphological change in microglia after experimental diffuse brain injury^23^. Rod microglia and rod microglia trains (multiple connected rod-microglia spanning > 100 µM) form predominantly in the primary somatosensory cortex barrel fields (S1BF) after experimental diffuse brain injury. Inferring function from form, rod microglia may serve as the splinting of neuronal dendrites, forming a barrier to segregate healthy and damaged neurons, or facilitating microglia migration^24–26^. Moreover, the robust inflammation in the S1BF region, highlighted by rod-shaped microglia morphologies, accumulation of TSPO receptor, cytokine expression^27^ and neuronal atrophy, correlates to sensory behavior deficits^8,28^. In experimental diffuse TBI, the S1BF shows stark neuropathology, however the possibility exists for other cortical regions to harbor less overt neuropathology. Microglia morphology is ramified in the healthy brain and varies among brain regions (i.e cortex, hippocampus)^29^, which facilitates the maintenance of homeostasis through cellular surveillance. Microglial morphologic responses to pathophysiology are diverse, ranging from hyper-ramified to amoeboid shapes and are readily observed when proximal to severe injury. On the other hand, quantitative morphology analyses are necessary to distinguish the subtle microglia changes that more often accompany mild injury or at regions distant to severe injury^30–32^. Taking advantage of dynamic microglial responses to subtle disturbances in function, we hypothesized that microglia morphology would differ among cortical brain regions in the days following experimental diffuse brain injury. We employed sensitive and multiple quantitative methods (skeleton and fractal analysis) to discriminate microglia morphologies in order to test our hypothesis. The data in the present manuscript reveal diverse changes in microglia morphologies within the cortex after diffuse brain injury, and are progress toward a more comprehensive understanding of the diverse spatiotemporal cell responses to diffuse TBI.

## Methods

### Animals

Adult male Sprague-Dawley rats (350 to 375 g) were purchased (Envigo, Inc., Indianapolis, IN) and housed in a 12-h light cycle at a constant temperature (23 ± 2 °C) with food and water available *ad libitum*. All experiments were approved by and performed in accordance with the guidelines established by the University of Kentucky IACUC (Institutional Animal Care and Use Committee) and the NIH guidelines for the care and use of laboratory animals. Care was taken to minimize pain and discomfort. Data collection stopped at pre-determined final endpoints based on days post-injury for each animal. Pre-determined exclusion criteria included excluding animals if post-operative weight decreased by 15% of pre-surgical weight. No rats were excluded from this study. Randomization of animals was achieved by assigning animals to treatment groups before the initiation of the study to ensure equal distribution across groups. All analyses were conducted by investigators blinded to the treatment groups.

### Experimental Diffuse Brain Injury: Midline Fluid Percussion Injury

Rats were subjected to either sham operation or midline fluid percussion injury (mFPI) as previously described^23,26,33^ and reviewed briefly here. Rats were anesthetized with 5% isoflurane delivered in surgical grade air prior to the surgery and maintained at 2.5% isoflurane via nose cone. While anesthetized, body temperature was maintained using a Deltaphase® isothermal heating pad (Braintree Scientific Inc., Braintree, MA). In preparation for the mFPI procedure, anesthetized rats were placed in a stereotaxic frame and a 4.8-mm outer diameter circular craniotomy was performed, centered on the sagittal suture midway between bregma and lambda (approximately at −2.0 mm from bregma). During this process, great care was taken not to disrupt the underlying dura or superior sagittal sinus. A skull screw was then secured in a 1-mm hand-drilled hole. An injury hub was fabricated from the female portion of a Luer-Loc needle hub, which was cut, beveled, and scored to fit within the craniotomy. The injury hub was fixed over the craniotomy using cyanoacrylate gel and methyl-methacrylate (Hygenic Corp., Akron, OH). The incision was sutured at the anterior and posterior edges and topical Lidocaine ointment was applied. Animals were recovered in a warmed holding cage and monitored until ambulatory. Diffuse brain injury was induced in rats 60 to 90 min after surgical preparation. For injury, rats were re-anesthetized with 5% isoflurane in 100% O_2_ and the dura was inspected through the injury-hub assembly. The hub assembly was filled with saline and attached to the male end of the fluid percussion device (Custom Design and Fabrication, Richmond, VA). Upon return of reflexive responses, the injury (1.9–2.0 atm) was administered by releasing the pendulum onto the fluid-filled cylinder. Rats were monitored for the presence of a forearm fencing response (one forelimb flexed, other extended) and the return of the righting reflex (4-10 min) as indicators of injury severity^30^. For the sham procedure, rats were connected to the fluid percussion injury device, but the pendulum was not released; sham-injured animals recovered the righting reflex within 15 seconds. The injury hub assembly was removed, the integrity of the dura was observed, and bleeding was controlled prior to the incision being stapled. Recovery was monitored postoperatively for up to three days, with no overt differences (e.g. weight, movement, grooming) observed between animals. A sham procedure was carried out for each time point (1, 7, and 28 days post-injury) and pooled to achieve a sham sample size of three for statistical comparisons.

### Immunohistochemistry and imaging

**Figure 1:**
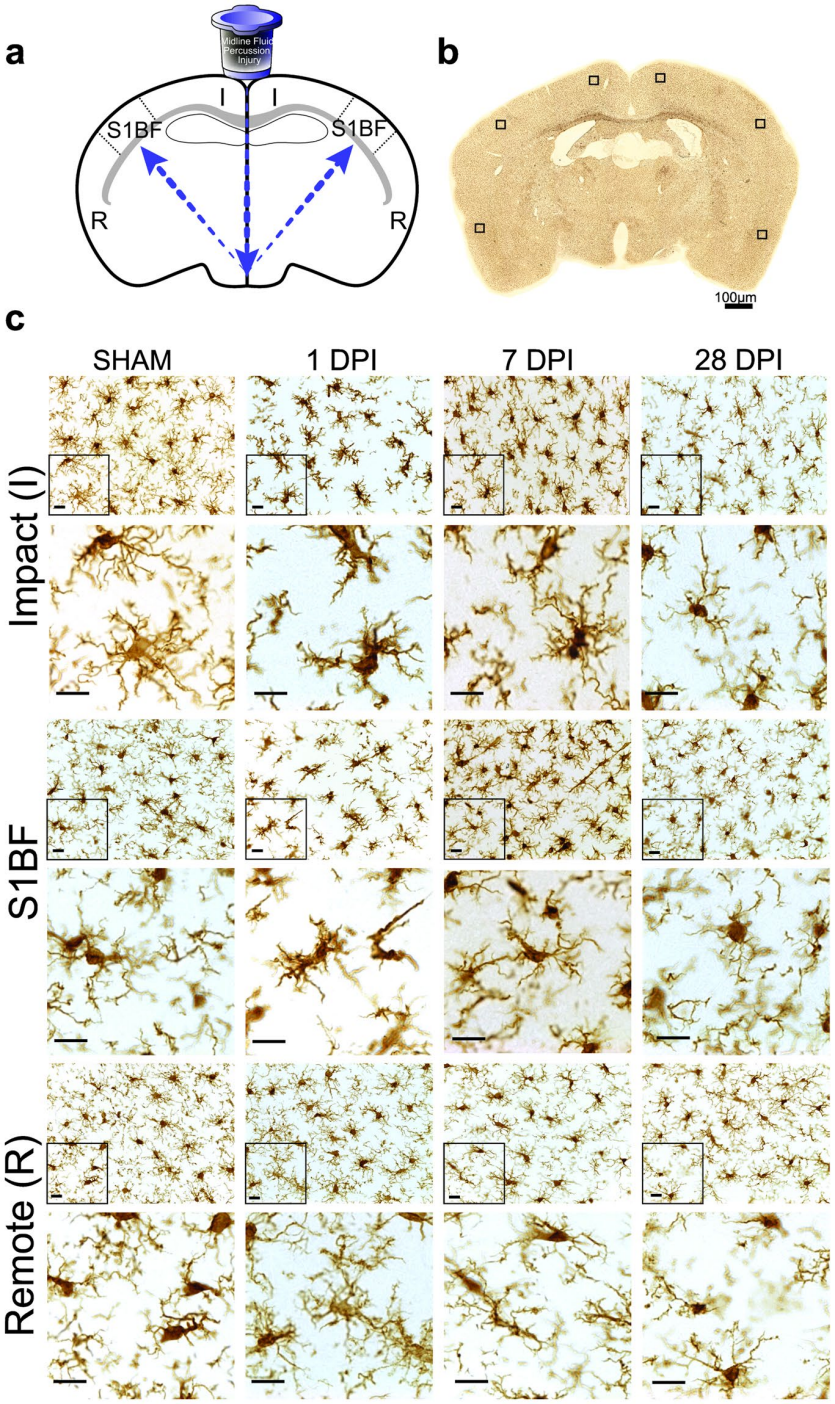
Iba1 stained microglia in cortex regions after midline experimental diffuse brain injury and days of recovery. (**a**) Schematic drawing depicting the midline fluid percussion injury (mFPI) delivered to animals and the brain regions (impact, somatosensory barrel field [S1BF], and remote) imaged within cortical layers I-III. The arrows illustrate the percussive force delivered by the mFPI model. (**b**) Approximate imaging regions per histological section. (**c**) Iba1 immunostaining of impact, S1BF, and remote brain regions in sham animals and 1, 7, and 28 days post-injury (DPI). Cropped photomicrographs corresponding to the highlighted square are provided to show microglia morphology detail. All quantitative skeleton analysis was carried out on full sized photomicrographs while fractal analysis was carried out on single cells. (Scale bars = 10 µm unless otherwise noted).

Immunostaining to identify microglia was carried out on tissue sections as previously described^23^. Animals were euthanized at designated time points (1, 7, or 28 days; n = 3 for each time point) post-injury or sham operation (sham; n = 3); tissue was first perfused with saline followed by 4% paraformaldehyde/PBS. Brain tissue was removed and fixed in a 4% paraformaldehyde followed by 15% sucrose solution for 24hr prior to being transferred to fresh fixative solution and sent for histological processing by Neuroscience Associates Inc. (Knoxville, TN, USA). The brains were embedded into a gelatin matrix where they could be frozen and sectioned from one solid block (MultiBrain® Technology, NeuroScience Associates, Knoxville, TN). Sections of 40 μm thickness were taken in the coronal plane, wet-mounted on 2% gelatin-subbed slides, and stained for microglia using ionized calcium binding adaptor molecule (Iba1) primary antibody and 3,3′-Diaminobenzidine (DAB) visualization (NeuroScience Associates, Knoxville, TN). Digital imagining was carried out on an Olympus AX-80 microscope and attached DP-70 digital camera (Olympus America Inc., Center Valley PA) using a 40x objective. Three coronal brain sections per animal (between bregma −0.45 and −1.85) were imaged twice, once in each right and left hemisphere, for each region of interest and at each time point (sham, 1, 7, or 28 days post-mFPI) to yield six digital photomicrographs per animal and region for analysis. The regions imaged were: near to the impact site (retrosplenial cortex region), primary somatosensory barrel field (S1BF) cortex, and a site remote to the impact (entorhinal cortex). The three regions of interest are depicted in Fig. 1a and b. Microglia were analyzed for changes in ramified morphology after experimental diffuse brain injury using computer-aided skeleton analysis methods^34^. Photomicrographs of microglia at 40X magnification in the impact (I), S1BF, and remote (R) regions over days post-injury (DPI) are shown Fig. 1c. All sections were uniformly stained and clear of background. Cropped photomicrographs are included below each raw image to better visualize microglia morphology detail; however, data were collected from uncropped images. All photomicrographs used for analysis were obtained from a single focal plane, in which microglia and processes are randomly oriented. Random orientation approximates the nucleator method for estimating three dimensional data from two dimensional images, when populations of cells are analyzed^35^, however additional data could be obtained from image stack visualization in future studies.

### Skeleton Analysis

**Figure 2:**
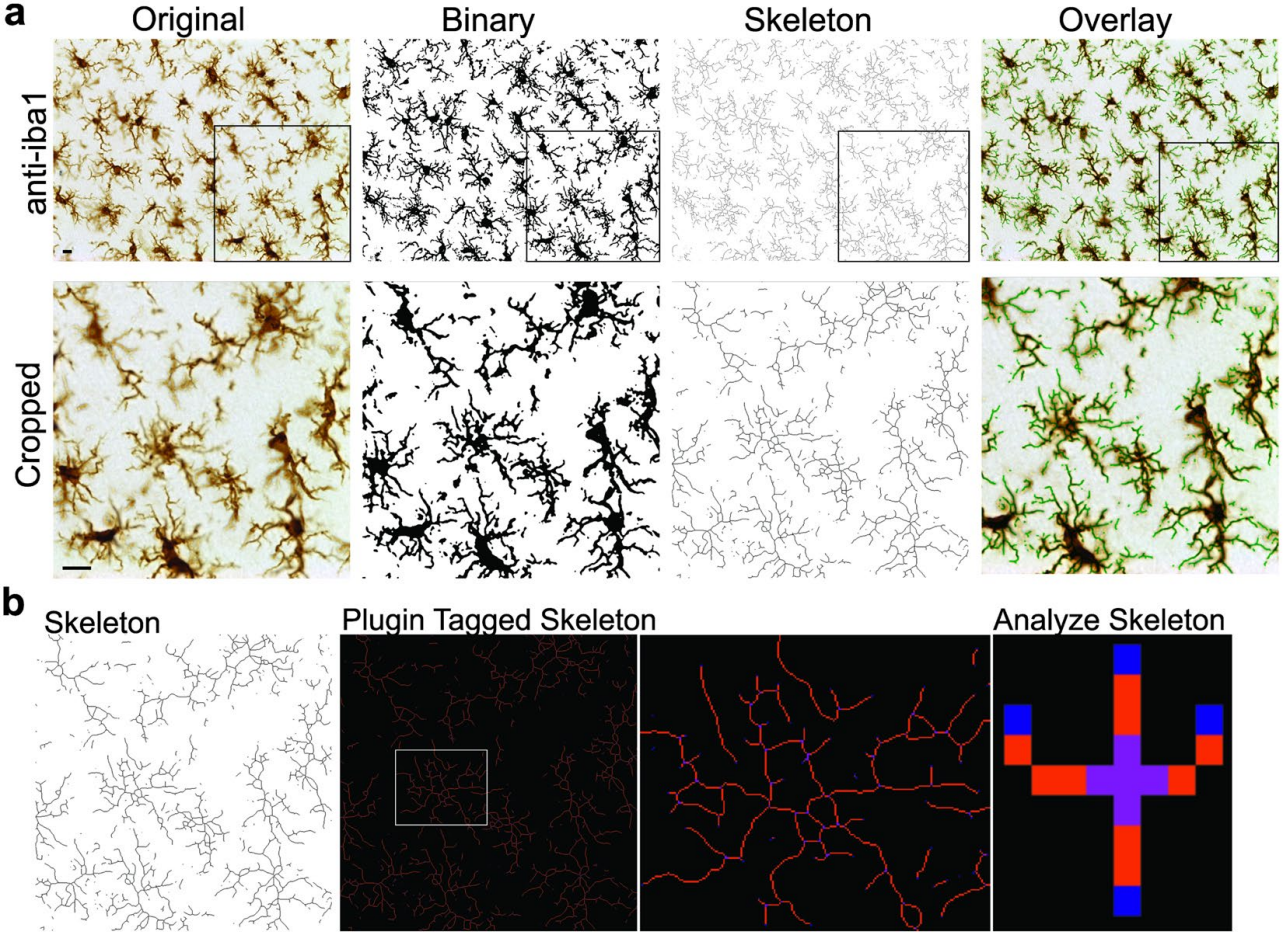
Skeleton analysis of microglia morphologies in Iba1 stained tissue. (**a**) The process to prepare photomicrographs for skeleton analysis. Original photomicrographs were subjected to a series of uniform ImageJ plugin protocols prior to conversion to binary images; binary images were then skeletonized. An overlay of a resulting skeletonized image (in green) and original photomicrograph shows the relationship between skeleton and photomicrograph. Cropped photomicrographs (below) show additional detail and all skeleton analysis was completed on full sized photomicrographs (Scale bar = 10 µm). (**b**) The skeletonized images are processed using the Analyze Skeleton plugin (maintained here: http://imagej.net/AnalyzeSkeleton) to identify and tag skeletonized processes as orange, endpoints as blue, and junctions as purple. The tagged data are then organized and data output summarized.

A ramified cell is one that has a complicated network of processes that originate from the cell soma. A change in microglia ramification indicates a microglial response to an altered physiologic status, in this case induced by mFPI. ImageJ software (National Institute of Health, https://imagej.nih.gov/ij/ ^34^) and appropriate plugins (i.e. FFT bandpass filter, unsharp mask and close) were consistently used prior to converting all photomicrographs to binary and skeletonized images. In addition to creating skeletonized images, cell somas were manually counted for each photomicrograph. The Analyze Skeleton Plugin (developed by and maintained here: http://imagej.net/AnalyzeSkeleton31) was then applied to the skeleton image which tags skeletal features relevant to microglia ramification: slab voxels (orange, process length) and endpoints (blue). Figure 2 illustrates the workflow process to convert an entire original photomicrograph to a plugin tagged image (original, binary and skeleton); cropped images and an overlay of skeleton to original image is provided for detail and to illustrate that skeletons are representative of the original image. We summarized the number of process endpoints and length from the Analyze Skeleton plugin data output and normalized all data by the number of microglia cell somas in each image to calculate the number of microglia endpoints/cell and microglia process length/cell. In Table 1, we summarize skeleton analysis measures (endpoints and process length/cell) in terms of measure, unit, range, scale, and interpretation.

**Table 1:** Summary of microglia morphology measures.

	Measure	Unit	Range	Scale	Sampling	Interpretation
Process length^22^	Summed	µm/cell	Continuous	Photomicro-graph	6 photomicrographs/animal	Cell ramification
Process endpoints^22^	Summed	#/cell	Continuous	Photomicro-graph	6 photomicrographs/animal	Cell ramification
Fractal Dimension^36,37^	$\mathrm{regression}\,\mathrm{slope}[\frac{In(N)}{In(\varepsilon )}]$	D_B_	1-2	Individual cell	24 cells/animal	Cell complexity
Span Ratio^38^	$\frac{{\rm{convex}}\,{\rm{hull}}\,{\rm{eclipse}}\,{\rm{longest}}\,{\rm{length}}}{{\rm{convex}}\,{\rm{hull}}\,{\rm{eclipse}}\,{\rm{longest}}\,{\rm{width}}}$	Ratio	0-1	Individual cell	24 cells/animal	Cell shape
Density^38^	$\frac{\#\,\mathrm{of}\,\mathrm{pixels}\,\mathrm{within}\,\mathrm{cell}\,\mathrm{outline}}{\mathrm{area}\,\mathrm{of}\,\mathrm{convex}\,\mathrm{hull}}$	$\frac{\#\mathrm{of}\,\mathrm{pixels}}{{\rm{area}}}$	0-1	Individual cell	24 cells/animal	Cell Size

### Fractal Analysis using FracLac for ImageJ

We extended our computer-aided morphologic analysis after diffuse brain injury to include fractal analysis (FracLac for ImageJ) which quantifies, among other things, cell complexity (fractal dimension, D_B_), density, and span ratio^36,37^. FracLac analysis is typically applied to single cells as opposed to the entire image field used for skeleton analysis. Four microglia were randomly chosen for fractal analysis (using a grid and random number generator) within each photomicrograph (6 photomicrographs per animal) for a total of 24 cells analyzed per animal in each region. Analysis included fractal dimension, span ratio, and density. Individual microglia were first made binary through a similar process as described for skeleton analysis. The additional structures that abut and surround each cell were excluded from analysis by manual deletion using a digitizing tablet and ImageJ. Binary images were then converted to outlines using ImageJ. We summarize all fractal analysis measures (fractal dimension, span ratio, and density) in terms of measure, unit, range, scale, and interpretation in Table 1.

**Figure 3:**
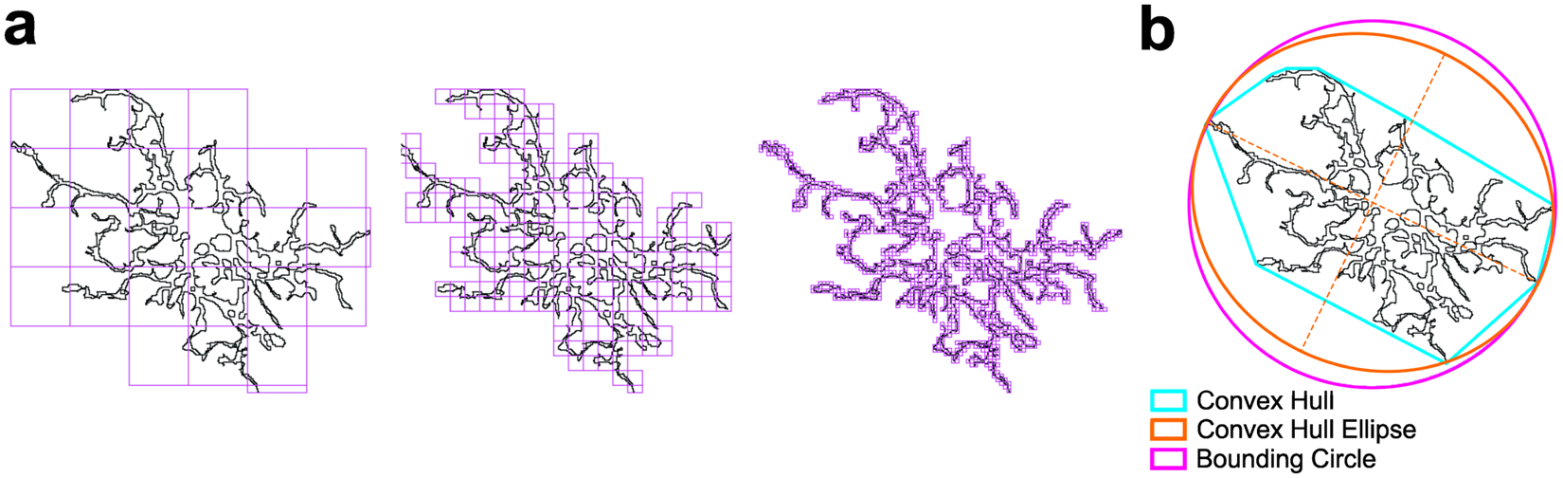
FracLac for ImageJ to quantify cell complexity and shape. (**a**) Illustration of FracLac box counting method to derive fractal dimension calculations of a microglia outline. Shape detail is quantified as scale increases, represented by pink boxes. Box counting equation is summarized in Table 1. (**b)** Schematic of the convex hull (blue), bounding circle (pink) and convex hull ellipse (orange) with accompanying longest length and width (dashed lines) necessary for calculating microglia span ratio and density (Table 1).

Fractal dimension is a measure of microglia complexity, which quantifies each cell’s contour bounded by the endpoints and process lengths. FracLac for ImageJ calculates the microglia fractal dimension (D_B_) for each cell using a box plot protocol that determines the amount of pixel detail with increasing scale, where N = the number of pixels or “detail” at a particular scale (ɛ) (see Fig. 3a and Table 1). These calculations and relationships are best summarized in the reference guide provided for FracLac for ImageJ http://rsb.info.nih.gov/ij/plugins/fraclac/FLHelp/Introduction.htm and additional associated references^32–34^. In summary, a microglia is more complex as the amount of detail grows quickly with increasing scale or, as D_B_ approaches 2.

We also summarize additional measures of microglia morphology related to cell shape: span ratio and density^34^. Such measures use the FracLac generated “convex hull” which circumscribes each microglia outline with a polygon and a circle that bounds the convex hull. An example of the convex hull, eclipse and bounding circle is provided in Fig. 3b. Span ratio is the ratio of the convex hull ellipse’s longest length to longest width. Density is the ratio of the number of pixels encompassed by the cell outline to the area (in pixels) of the convex hull. Span ratios are indicators of cell shape, whereas density is an indicator of cell size.

### Statistical Analysis

Data are shown as mean ± SEM and analyzed using GraphPad Prism 6 with statistical significance assigned when p < 0.05, unless otherwise indicated. Differences in cell counts/field, endpoints per cell, summed process length, fractal dimension, span ratio, density, and a spatial-temporal interaction between region and time post-injury were analyzed using a two-way analysis of variance (ANOVA) followed by a two-tailed Tukey’s multiple comparisons test.

## Results

### Microglia are de-ramified in the impact and S1BF regions after diffuse brain injury

**Figure 4:**
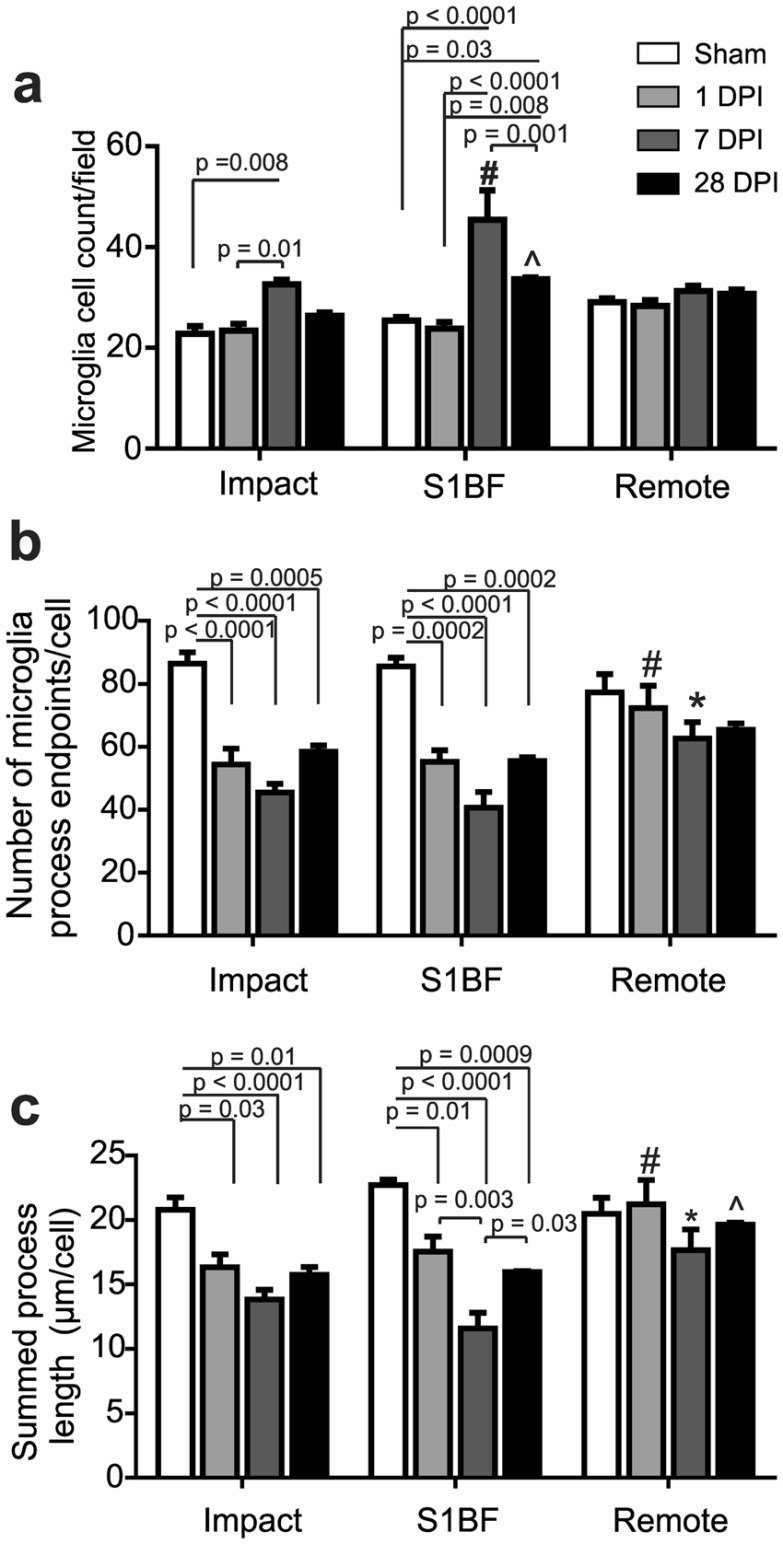
Microglia ramification is different in brain regions following experimental diffuse brain injury. (**a**) Summary data (mean and SEM) and statistical analysis (two-way ANOVA) of microglia cell counts/field in brain regions following diffuse brain injury (image n = 6/group, animal n = 3/group). At 1, 7, and 28 days post-injury (DPI), microglia counts are higher than sham within regions (F_(2,24)_ = 9.07, *p = *0.001) and with time (F_(3,24)_ = 21.50, *p* < 0.0001) with significant interaction (F_(6,24)_ = 5.08, *p* = 0.002). Post-hoc analyses are reported within the figure (^#^ *p* = 0.003 vs impact and ^^^ *p* < 0.003 vs. remote). (**b)** Summary data and statistical analysis of microglia endpoints/cell at 1, 7, and 28 DPI (n = 3/group). The number of microglia endpoints/cell is different than sham after mFPI (F_(3,24)_ = 33.67, *p* < 0.0001). There are fewer endpoint/cell in the impact and S1BF regions at all DPI, but not at the remote region (post hoc are summarized in figure). The number of endpoints/cell was also different among brain regions at time points (F_(2,24)_ = 6.56, *p* = 0.005) with post-hoc analysis reported in figure (1DPI ^^^ *p* < 0.05 vs S1BF and Impact; 7DPI ^#^ *p* < 0.05 vs Impact and S1BF). There was a significant interaction effect (F_(6,24)_ 3.17, *p* = 0.02) **c)**. Summary data and statistical analysis of microglia process length/cell in brain regions at 1, 7, and 28 DPI (n = 3/group). Summed process length is different than sham in the days following mFPI (F_(3,24)_ 21.78, *p* < 0.0001). Process length is decreased at all DPI in the impact and S1BF regions, but not the remote region (post hoc analyses are summarized in the figure). Microglia process length/cell was also different among brain regions at time points (F_(2,24)_ 9.99, *p* = 0.0007) with post hoc analysis reported in figure (1DPI ^^^ *p* < 0.05 vs S1BF and Impact; 7DPI ^#^ *p* < 0.05 vs Impact and S1BF; 28DPI ^^^ *p* = 0.04 vs Impact). There was a significant interaction effect (F_(6,24_) = 3.07, *p* = 0.02).

Figure 1b illustrates microglia photomicrographs in each region. We first quantified the number of microglia cells in each field in order to normalize skeleton analysis data. In this case, we report significant differences in cell counts/field and a spatial-temporal interaction between region and time post-injury (2way ANOVA: region: F_(2,24)_ = 9.07, *p* = 0.001; time: F_(3,24)_ = 21.50, *p* < 0.0001; interaction: F_(6,24)_ = 5.08, *p* = 0.002). Microglia cell count was increased in the impact (7 DPI) and S1BF regions (at 7 and 28 DPI; all post-hoc results are reported within Fig. 4a). We next measured changes in microglia ramified morphology in brain regions and in the days following mFPI by quantifying the number of microglia endpoints and process length per cell. Microglia endpoints differed according to region and time (2way ANOVA: region: F_(2,24)_ = 6.56, *p* = 0.005; time: F_(3,24)_ = 33.67, *p* < 0.0001; interaction: F_(6,24)_ = 3.17, *p* = 0.02). Post-hoc analyses reveal that there were fewer microglia process endpoints at all DPI when compared to sham in both impact and S1BF regions, but not in the remote region. All post-hoc results are reported in Fig. 4b. Similar to endpoints/cell, summed process length/cell differed according to region and time (2way ANOVA: region: F_(2,24)_ = 9.99, *p* = 0.007; time: F_(2,24)_ = 21.78, *p* < 0.0001; interaction: F_(6,24)_ = 3.08, *p* = 0.02). Process length/cell was significantly less than sham in the impact and S1BF regions at all DPI but, again, not in the remote region. All post-hoc results are reported in Fig. 4c. In summary, skeleton analysis of microglia morphologies revealed that microglia become de-ramified (fewer and shorter processes per cell) in the impact and S1BF regions as early as 1 DPI, which persisted until 28 DPI—the end of the study. However, that microglia are not de-ramified in the region remote to the impact site suggests that microglia de-ramification is not universal throughout the diffuse-injured brain.

### Microglia complexity and shape differs by region in the days following diffuse brain injury

**Figure 5:**
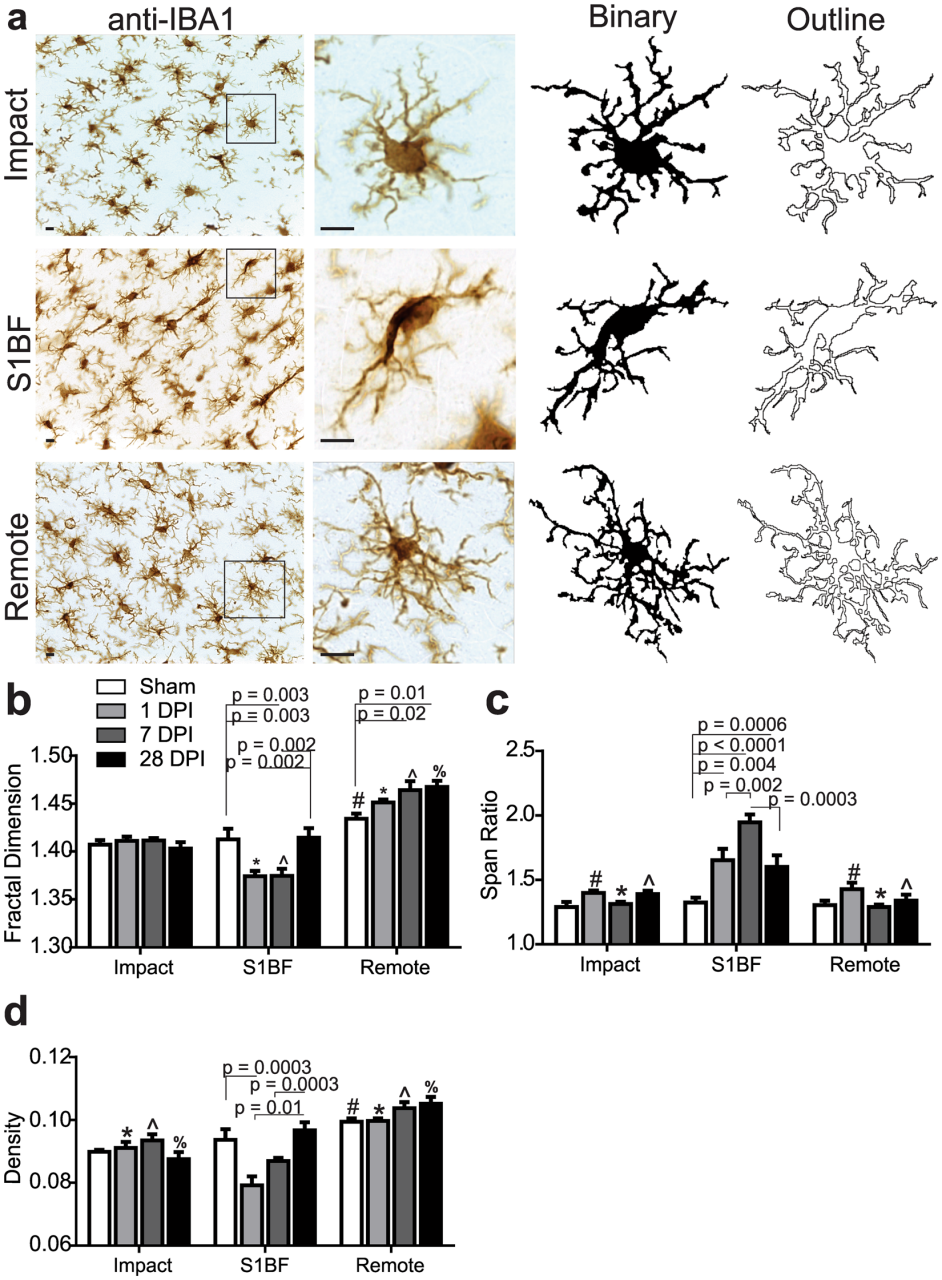
Microglia complexity, elongation, and size are different in brain regions following experimental diffuse brain injury. (**a**) Example photomicrographs and cell outlines of Iba1/DAB microglia in impact, S1BF, and remote regions; scale bar = 10 µm. (**b**) Summary data and statistical analysis of fractal dimension at 1, 7, and 28 DPI. Microglia fractal dimension was different than sham post-mFPI within brain regions (F_(3,24)_ = 2.99, *p* = 0.05), decreased in the S1BF region after 1 DPI and 7 DPI, and increased in the remote region at 7 and 28 DPI. Microglia fractal dimension was also different according to time (F_(2,24)_ = 84.07, *p* < 0.0001). There was a significant interaction effect (F_(6,24)_ = 5.53, *p* = 0.0001). All post-hoc analyses are reported in the figure (Sham: ^#^ *p* < 0.01; 1DPI: ^*^ *p* < 0.05 vs impact and S1BF; 7DPI: ^^^ *p* < 0.05 vs Impact and S1BF; 28DPI: ^%^ *p* < 0.05 vs Impact and S1BF). **c)** Summary data and statistical analysis of span ratio at 1, 7, and 28 DPI. Microglia span ratio was different than sham within brain regions in the days following mFPI (F_(3,24)_ = 10.43, *p* < 0.0001), elongated in the S1BF region after 1, 7 and 28 DPI. Microglia span ratio was also different between brain regions (F_(2,24)_ = 43.15, *p* < 0.0001). All post hoc analyses are reported in the figure (1DPI: ^#^ *p* < 0.05 vs S1BF; 7DPI: ^*^ *p* < 0.0001 vs S1BF; 28 DPI: ^^^ *p* = 0.05 vs S1BF). There was a significant interaction effect after mFPI (F_(6,24)_ = 8.9, *p* < 0.0001). (**d**) Summary data and statistical analysis of density at 1, 7, and 28 DPI. Microglia density was different than sham within brain regions after mFPI (F_(3,24)_ = 5.28, *p* = 0.006). Microglia density was decreased when in the S1BF region at 1 DPI when compared to sham but increased at 28 DPI. Microglia density was also different between brain regions (F_(2,24)_ = 46.54, *p* < 0.0001). There was a significant interaction effect after mFPI (F_(6,24)_ = 6.11, *p* = 0.0005). All post hoc analysis reported in figure (Sham: ^#^ *p* < 0.01; 1DPI: ^*^ *p* < 0.05 vs S1BF and Remote; 7DPI: ^^^ *p* < 0.01 vs S1BF and Remote; 28DPI: ^%^ *p* < 0.05 vs S1BF and Remote). All data are mean ± SEM with two-way ANOVA analysis.

Examples of microglia (made binary and outlined) in each brain region are shown in Fig. 5a. Application of FracLac for ImageJ to microglia outlines resulted in fractal dimensions that ranged from 1.374 to 1.457 (available range is 1-2) with the lowest value occurring in the S1BF region and the highest in the remote region. Two-way ANOVA analysis identified a spatiotemporal relationship in microglia fractal dimension post-injury (time: F_(3,24)_ = 2.99, *p* = 0.05; region: F_(2,24)_ = 84.07, *p* < 0.0001; interaction: F_(6,24)_ = 6.53, *p* = 0.0003; all post-hoc results are summarized in Fig. 5b). Microglia complexity is unchanged from sham in the impact region whereas, in the S1BF region, cell complexity was low at 1 and 7 DPI and recovered to sham condition by 28 DPI. In the remote region, cell complexity steadily increased from sham condition until 28 DPI, when the study ended. The wide range of fractal dimension scores suggests that complexity of microglia shapes is diverse throughout the diffuse-injured brain.

Using FracLac for ImageJ, we investigated additional measures of microglia morphology related to cell shape: span ratio and density. Span ratio is a measure of microglia elongation and therefore relevant to our previous detection of rod microglia in the S1BF region after mFPI. Span ratio was significantly increased post-injury, however, only in the S1BF region and peaking at 7 DPI (time: F_(3,24)_ = 10.43, *p* = 0.0001; region: F_(2,24)_ = 43.15, *p* < 0.0001; interaction: F_(6,24)_ = 8.89, *p* < 0.0001; post-hoc results reported in Fig. 5c). Density is used to report on microglia size. We illustrate that the most robust changes to microglia size also occurred in the S1BF region at 1 DPI and was recovered by 28 DPI post-injury (time: F_(3,24)_ = 5.28, *p* = 0.006; region: F_(2,24)_ = 46.54, *p* < 0.0001; interaction: F_(6,24)_ = 6.11, *p* = 0.0005; post-hoc results are reported in Fig. 5d).

### Combined Skeleton Analysis and Fractal Analysis reveal diverse microglia morphologic responses after diffuse brain injury

**Figure 6:**
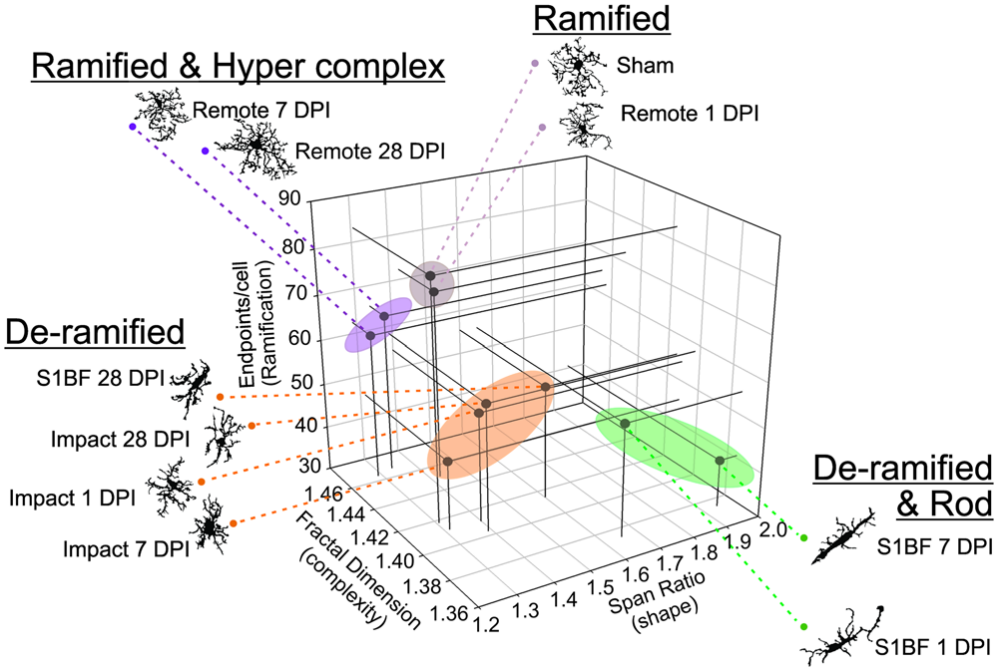
Diverse microglia morphologies in the days following diffuse brain injury. Fractal dimension (D_b_), endpoints/cell, and span ratio data were averaged for each region and time for Pearson’s correlation (n = 10). The figure summarizes the relationship between all three variables with exemplars of microglia for data points. Fractal dimension (D_B_) is inversely related to span ratio (r = −0.69, p = 0.03) (r = 0.55, p = 0.10); endpoints/cell is not significantly correlated to span ratio (r = 0.58, p = 0.08). Four groupings emerge: Ramified, ramified and hyper-complex, de-ramified and de-ramified and rod. Fractal dimension was inversely related to span ratio but not endpoints/cell.

Last, we present the relationship between skeleton and fractal analysis data by plotting endpoints/cell, fractal dimension, and span ratio; data were averaged for each time and region. These three measures were chosen as single variables to represent cell ramification, complexity, and shape, respectively. Including all variables would be redundant as process length and endpoints/cell were strongly correlated (r = 0.95, p < 0.0001) as were density and fractal dimension (r = 0.95, p < 0.0001); span ratio and circularity were inversely correlated (r = −0.99, p < 0.0001). Fractal dimension was inversely correlated to span ratio (r = −0.69, p = 0.03) but not to endpoints/cell (r = 0.55, p = 0.10); endpoints/cell was not significantly correlated to span ratio (r = 0.58, p = 0.08). Plotting these three parameters on a scatterplot identified groups of cells with particular morphologic responses in the days after diffuse brain injury and have categorized microglia morphologies as ramified, ramified & hyper-complex, de-ramified, and de-ramified & rod (Fig. 6).

## Discussion

Microglia are in close communication with immediately surrounding cells and are endogenous reporters of altered or disrupted neuronal and glial function. It follows that microglia morphology, if quantified using sensitive methods, could provide insight into neuropathology of discrete regions after diffuse brain injury. On this basis, our purpose was to define the spatiotemporal changes in microglia morphology throughout the 28 days following rat mFPI as a first step in exploiting the microglia’s potential to reflect altered brain physiology. Using multiple quantitative assessments of microglia morphology we report that microglia morphology was different from sham in all three imaged regions at 28 days post-injury. We have summarized these diverse morphologies as: ramified; ramified/hyper-complex, de-ramified, and de-ramified/rod morphologies. That microglia become de-ramified post-injury is commonly reported while cell hyper-ramification or increased complexity is observed less frequently. Our data illustrate that microglia responses post-mFPI includes a wide spectrum of morphologies and likely reflects an equally diverse neuronal response. At this time, however, we make no claim to the temporal relationship or transitions between microglia morphologies nor the direct relationship to neuronal function. However, in future research, it is possible that microglia morphologies will indicate neuronal and glial function or dysfunction after experimental diffuse brain injury.

Neuroinflammation is a secondary injury process that can extend the primary diffuse brain injury. If properly identified and understood, in terms of key components and timing, this secondary injury is a treatment target to attenuate pathophysiology that contributes to neurological symptoms (for a review please see^39^). As an added level of complexity, microglia mediated neuroinflammatory responses can be beneficial or detrimental, as demonstrated by the simultaneous pro-inflammatory and pro-repair mechanisms of inflammation (for a recent review please see^40^). In light of this duality, it becomes imperative to understand the diversity of microglia responses post-injury in order to provide precision of care both in therapeutics and timed delivery. Although neuronal dysfunction is not directly studied, our data illustrate that a broad diversity of microglia responses are present in the cortex over 28 days post-mFPI, which we interpret to correspond with changes in neuronal activity. When assessed qualitatively, microglia morphology appeared similar throughout the brain, with exception of the rod-like microglia present in the S1BF. While it was reasonable to postulate changes in microglia morphology would be present in regions nearest to the impact region, a finding of this study, no changes in microglia ramification were expected in the remote region. Therefore our data highlight the need for an objective, quantitative analysis to detect and report with accuracy diverse morphologies in conditions where disease and injury may result in subtle pathophysiology. A next step is to link the defined microglia morphologic states to pro-inflammatory versus pro-repair and mechanisms of inflammation.

Much focus remains on microglia de-ramification as the primary indicator of a microglial response or “activation” following injury. However, in addition to de-ramification, our analysis revealed that microglia become progressively more complex and cell density increases over 28 days post-injury in the remote brain region. We have categorized these cells as ramified and hyper-complex. Others have identified regions that contain hyper-ramified morphologies *in situ*, which may be similar to our hyper-complex category. Hyper-ramified morphologies were previously identified in female mouse brain tissue^41,42^ or in mice with chronic stress^43–45^. In chronic stress, a hyper-ramified/hyper-complex morphology has not been correlated to neuropathological indicators of injury, such as IL1-β, CD68, or caspase, but instead increased expression of β1-integrin^44^. In this case, hyper-ramified morphologies represent a stress-induced disruption of the extracellular matrix. In contrast, the hyper-ramified response has also been described as an indicator or early response to injury^22,46^. It remains unclear if the hyper-ramified/hyper-complex morphology should be considered as part of the injury response, a change in the extracellular milieu, or in response to intense neuronal activity. For example neurotransmitter agonists NMDA, AMPA, kainite, glutamate, GABA^12,15^ and adenosine (via A3 receptor)^47^ elicit an ATP-induced increased ramification. We propose that hyper-complex microglia in the remote region represent a combination of protection against a spreading wave of neuropathology or downstream second-order neuropathology from remodeled circuits which may result in increased neuronal excitatory signaling.

We employed fractal analysis of single cells in order to report on cell complexity, the level of detail in the microglia shapes, the span or elongation of microglia, and cell size. Microglia complexity (fractal dimension values) were in line with previous findings which suggest our use of FracLac was adequate^32,33,40,48^ and may be compared with other microglia quantification studies when studying similar brain regions^26^. As an added benefit, FracLac calculates additional representations of morphologic change for consideration, such as cell shape and cell size. Our data summary of microglia span ratio, a measure of cell shape/elongation, validates our previously reported findings and reiterates that de-ramified rod microglia are present only in the S1BF region, which peaked at 7 DPI^23^. Moreover, we confirm previous findings that rod microglia are elongated in roughly a 1.8:1 length:width ratio^22,23^.

Alternate methods can quantify microglia morphologies and have been applied in focal TBI models^49–51^. In these studies, microglia were classified *a priori* into three categories based on morphology: ramified, hypertrophic, and bushy. In contrast, the analyses used in this study provide continuous data (rather than categorical) to describe the broad continuum of microglia morphologic changes. ImageJ methods (FracLac and/or Skeleton analysis) have been used to detect subtle injury and/or neuronal dysfunction as well as gross injury such as closed-head impact model of TBI^48^ and ischemic stroke^22,30^. As an added benefit, ImageJ based analysis (skeleton analysis and FracLac for ImageJ) is free, simple to use, rapid, and easily complements data collection using proprietary software. The ImageJ skeleton analysis method is high throughput when compared to cell-by-cell analysis and may be employed as a screening tool to identify and screen areas of neuronal dysfunction or prior to more in-depth analysis. It is our goal that with ease of use, the assessment of microglia morphology will be incorporated into additional studies, lead to a better detection and therefore understanding of neuronal dysfunction and death, and understanding of diverse microglial functions across a continuum of brain injury—mild to severe.

Novel to this study, we were able to stratify microglia morphologies by combining three quantitative outcome variables acquired via both skeleton and fractal analysis to reveal four categories of cell morphologies: ramified, ramified and hyper-complex, de-ramified, and de-ramified and rod. The variables used, endpoints, span ratio, and fractal dimension were chosen as non-redundant (not correlating) representations of three morphology parameters: ramification, shape, and complexity. The use of either the skeleton or fractal analysis method alone was insufficient to separate these distinct morphologic categories.

Currently, it is difficult to identify regions of neuropathology after diffuse TBI, because of non-obvious histological changes and the existence of multiple pathological axonal phenotypes^8^. For this study, clinically relevant diffuse TBI by midline fluid percussion injury occurs without cavitation, as seen with focal and mixed experimental models of TBI^52,53^. Quantifying microglia morphology is a relevant method and useful tool in the discovery of multi-focal and yet apparently uninjured regions of neuropathology after diffuse brain injury. As a continuous variable, the degree of cell ramification, complexity, shape, and size can be correlated to either microglia function or parenchymal responses, such as neuron or glial functions, once discovered. Such correlations are the important next step in the discovery of subtle neuronal dysfunction in the days following diffuse brain injury and the tracking of recovery. Our findings may be relevant to the investigation of progressive disease conditions, particularly those associated with aging. The highly available and simple to employ quantitative tools are likely to be sufficiently sensitive to identify hyper-ramified or hyper-complex morphologies as early detection of injury and/or disease. Continued investigations will bring to light microglia morphologies with function in relation to neuronal or glial activity, which together can inform health, disease, or injury in clinical management.
